# Central Midwives Board for Scotland

**Published:** 1920-11-27

**Authors:** 


### Central Midwives Board for Scotland.

At a meeting of the Board for the Hearing of Penal Cases (Sir J. Halliday Croom in the chair) appeared No. 1015, Annie Maria Quillan, 19 McDougall Street, Parkhead, Glasgow, in answer to charges of failure to attend on the mother and child during the puerperium period, as also failure to notify *ophthalmia neonatorum*, and other breaches of the rules.

Dr. Barbara Sutherland, Assistant to the Medical Officer of Health, Glasgow, and Miss Jane Corbett Baker, Health Visitor, Glasgow, appeared in support of the charges. The Board found the charges to be proved, and instructed the Secretary to remove her name from the Roll of Midwives and to cancel her Certificate.

At the same diet there appeared No. 2528, Mary Espenser, 20 Green Street, Calton, Glasgow, in answer to charges of failure to notify *ophthalmia neonatorum*, also failure to send for medical assistance in a case of excessive hæmorrhage, and other breaches of the rules.

Dr. Sutherland appeared in support of the charges.

The Board found the charges to be proved, and instructed the Secretary to remove her name from the Roll of Midwives and to cancel her Certificate.

